# Change of Mechanical Properties of Repair Mortars after Frost Resistance Rests

**DOI:** 10.3390/ma14123199

**Published:** 2021-06-10

**Authors:** Grażyna Łagoda, Tomasz Gajda

**Affiliations:** 1 Department of Bridges, Faculty of Civil Engineering, Institute of Roads & Bridges, Warsaw University of Technology, Al. Armii Ludowej 16, 00-637 Warsaw, Poland; g.lagoda@il.pw.edu.pl; 2 Bridge Department, Road and Bridge Research Institute, Instytutowa 1, 03-302 Warsaw, Poland

**Keywords:** mortar, frost resistance, cycling freezing and thawing

## Abstract

The use of repair mortars for concrete structures repair with no or limited resistance to the impact caused by freeze and thaw cycles is often the primary repair failure cause. This is particularly important in Poland. Due to the geographical location of the country, there is a large temperature difference between summer and winter. The number of passes through the threshold temperature of 0 °C throughout the year in the winter season exceeds 100. The article presents a comparison of the frost resistance results of tests of repair mortars. The first method was performed according to the Polish Guidelines (without the use of de-icing salts) and the second method according to PN-EN 1504-3 (with the use of de-icing salts). The results obtained were inconsistent in many areas. In particular, significant differences in the results for the change in compressive strength and the change in bending strength were observed. In the case of the frost resistance testing without the use of de-icing salts, a decrease in compressive strength was usually accompanied by a decrease in bending strength. In the case of frost resistance tests with the use of de-icing salts, an increase in the bending strength of mortars was observed (even by a dozen or so percent) with a decrease in the compressive strength of mortars (even by several dozen percent).

## 1. Introduction

Concrete structures are exposed to the impact of their environment through physical, chemical and biological processes. This also applies to concrete structure elements repaired with repair mortars which become an integral part of the concrete structure after placement. In order to determine the environmental effects, seven concrete exposure classes were introduced in the standards [1,2], corresponding to different conditions of use of particular structural elements. For bridges, the basic exposure classes considered for their design were XD3, XF3 and XF4 classes, with XF3 and XF4 classes being related to aggression caused by cyclic freezing and thawing: in the case of the XF3 class, by strong saturation with water without de-icing agents; and in the case of the XF4 class, by strong saturation with water with de-icing agents or seawater. Within the framework of European standardisation, new harmonised standards of the PN-EN 1504 series were introduced, including sheet PN-EN 1504-3 [3], which indicates test methods for assessing the performance of concrete repair mortars. Ageing tests in climate boxes were used to assess the future durability of concrete and concrete repair mortars. Standard PN-EN 1504-3 [3] introduced new ageing tests concerning thermal compatibility (determination according to [3]), including one test concerning frost resistance, evaluating resistance to cyclic freezing in a saturated NaCl solution and de-icing in water, according to PN EN 13687-1 [4]. In Poland, the test of frost resistance is used to assess functional properties. In the case of mortars, it is carried out in accordance with a modified test method currently described in PN-B-06265 [2], which is a national supplement to [1]. In the case of the national frost-resistance testing method, the tested mortar is subjected to cyclic freezing in air and thawing in water without the use of de-icing agents. The introduction of the harmonised standard PN-EN 1504-3 [3] dedicated to repair mortars made it possible for the producers of repair mortars to carry out an assessment of performance properties according to the requirements of the aforementioned standard, which in turn, if positive results are obtained, allow the product to be marketed in the European Union and to be CE marked without the need to carry out additional tests. The appearance on the Polish market of the first repair mortars complying with the requirements of PN-EN 1504-3 [3], where a new test method was used to evaluate their resistance to aggression caused by cyclic freezing and thawing gave rise to the idea of comparing them in terms of the results obtained with the method that was used nationally to evaluate repair mortars. Information from construction sites about failures of repair mortars marketed with CE marking, as well as the negative results obtained by these mortars in frost resistance tests according to the national method, made it necessary to perform comparative tests.

The aim of taking up the issue in question was to compare and analyse the ranges, methodology and results obtained during frost resistance tests regarding changes in the mechanical properties of repair mortars according to the method commonly used on the Polish market and the new method of frost resistance tests according to [3]. However, the basic question was whether the frost resistance testing method according to [3] is sufficient to properly evaluate repair mortars in terms of aggression caused by cyclic freezing and thawing.

## 2. Materials and Methods

### 2.1. Frost Resistance of Repair Mortars

The frost resistance of repair mortars, i.e., resistance to cyclic freezing and thawing processes that can cause both external and internal damage to the mortar, is often the decisive property that determines the mortar’s suitability for use. In Polish conditions, it is the factor that has the greatest influence on the durability of repairs made using repair mortars. This is directly related to Poland’s location in the transitional climate zone—between a moderate oceanic climate in the west and a moderate continental climate in the east. The consequence of this location is a large temperature difference between winter and summer—the temperature in summer reaches over 30 °C and in winter often falls below −20 °C, with frequent temperature transitions through 0 °C. In Polish conditions, the number of transition cycles through 0 °C, i.e., freezing and thawing cycles, per year is about 100. The lack of resistance to cyclic processes of repair mortar freezing and thawing is usually the main reason for the subsequent ineffectiveness of the repair carried out thereof.

In the case of the frost destruction process, we can say that it takes place on two levels: internal (internal destruction) and external (surface scaling), usually with the participation of de-icing agents. The frost destruction processes that occur in mortar are, in principle, the same as the frost destruction processes that occur in concrete, which have been described in detail in the literature, among others, in [5,6,7,8,9,10]. Over the years, many hypotheses have been formulated concerning the mechanisms of concrete frost destruction, reviewed by Marchand [11] and Pigeon [12], and summarised by Józwiak-Niedźwiecka in [13]. The basic mechanism of concrete damage caused by frost action is compared to the “closed container theory”. It refers to the fact that the specific volume of ice is about 9.3% larger than that of water, and an increase in pressure corresponds to the phase transition of water to ice in a closed container. The prerequisite is an adequate amount of water which should fill more than 91.7% of the container’s volume. Already at a temperature of −1 °C, the pressure reaches a value of about 10 MPa. This increase in pressure is high enough to destroy materials, especially those with porous structures, which have low tensile strength.

The person internationally recognized as the pioneer of frost destruction research is Powers, who in 1945, observed that the primary cause of frost destruction is hydraulic pressure [14], which increases during the ice formation process when water is “pushed out” of the pores where freezing has occurred. Freezing water increases in volume, causing unfrozen water to be “pushed” deep into the hollow pores. When the water flow path exceeds the critical distance, the pressure exerted by the water on the capillary walls exceeds the tensile strength of the grout, destroying its structure. The condition for the occurrence of critical pressure exceeds the boundary degree of water saturation. In order to eliminate the possibility of this type of destruction, additional pores in the concrete of adequate size and distribution must be introduced by means of aeration to compensate for this pressure. Additional pores are a kind of “buffer” into which water pushed out by the ice in the direction of the advancing icing is injected. It should be noted that there are two types of pressure in concrete when water freezes in the pores:

- Overpressure, caused by an increase in the volume of water when it freezes by about 9.3%;
- Negative pressure, caused by surface forces and consequent moisture migration in the material.

The hydraulic pressure in a pore system, in general, is a function [13] of the ice formation rate, the temperature drop rate, and the pore system and shape and associated resistance to water flow. The stresses in the concrete caused by the increase in the volume of freezing water are accompanied by stress concentrations caused by concrete heterogeneity. They occur at any temperature change because the aggregate and the grout or mortar are bodies that have different thermal properties [13]. The most destructive are repeated, fast freezing and thawing cycles [15]. Hydraulic pressure theory is particularly applicable to low-permeable, well-compacted and low-porous concretes, i.e., properties typical of repair mortars.

An interesting view on the damage to the concrete structure was presented by Cai and Liu [16]. They investigated the freezing of water in concrete’s pores subjected to a freeze–thaw cycle by tracking changes in the electrical conductivity of concrete at sub-zero temperatures. They found that the vast majority of damage to the concrete structure occurs at temperatures above −10 °C with the exception of high-strength concretes. In high-strength concrete, damage also occurs at temperatures below −10 °C. This is due to the finer pore structure.

However, the hydraulic pressure theory did not fully explain all the phenomena related to the damage of concrete caused by the action of negative temperature, so in 1953, Powers and Helmuth [17] presented the so-called osmotic pressure theory. The authors of the theory pointed out the different behaviour of non-aerated and aerated cement slurry samples. Aerated cement slurry samples increased in volume during freezing, while non-aerated slurry samples decreased in volume, i.e., shrank. According to the osmotic pressure theory, the freezing process begins in the larger pores called capillaries, and then as the temperature decreases, the pressure increases in the smaller pores. At a given temperature, there is a balance between the amount of ice and water in the capillary pores. After a certain amount of water has changed to ice, the concentration of the remaining solution increases and is high enough that without lowering the temperature, further change of water to ice does not occur. A difference in the concentration of the solution in the small pores is created, which induces osmotic pressure as the solution to equalize the concentrations by diffusing from smaller to larger pores with a higher solution concentration [13]. This is particularly noticeable when using de-icing agents, such as NaCl or CaCl_2_, as the salts cause osmotic pressure to occur and induce water movement towards the upper surface of the concrete, where freezing occurs and hydraulic pressure is marked [8].

The aforementioned hypotheses make the resulting frost damage dependent on non-equilibrium conditions, caused by the temperature drop below the free-water freezing point by generating internal pressure in the cement slurry. All subsequently developed hypotheses concerning the destructive action of cyclic freezing on the concrete structure are, to a greater or lesser extent, based on one of the three aforementioned theories [13]. The above-described mechanisms of destruction of the internal structure of concrete, i.e., the osmotic pressure mechanism in combination with the hydraulic pressure mechanism or the simpler mechanism of the so-called closed container, were used in the development of the test methods, including the test methods referred to in the national documents—the standard [2] or test procedures [18,19].

In the process of freezing and thawing to complete destruction, two stages of definitively different dynamics can be distinguished in the course of sample destruction. The initial stage is characterized by a significantly lower rate of material destruction process and lasts longer than the second stage. The two-stage course of the destruction process is observed regardless of the size and shape of samples and conditions of freeze–thaw cycles (one-sided or volumetric freezing, in air, in water or in aqueous solutions of salts of different concentrations) [20]. This phenomenon is also observed in the tests of repair mortars, where the stage of rapid destruction usually begins after about 150 cycles and lasts from a dozen to several dozen cycles. In the case of repair mortars, the visible beginning of the rapid destruction stage is the so-called swelling of the mortar manifested by an increase in weight and deformation of its shape as a result of the progressive internal destruction. According to [20], the first stage of slow destruction corresponds to the process of ageing and the accumulation of damage in the material in which the number of micro scratches per volume unit has not yet reached a critical value, after which the second stage of rapidly progressing, irreversible destruction of the material begins.

In the case of the process of frost destruction, taking place at the external level with the participation of de-icing agents, manifested by the scaling of the surface, the first studies, according to [13], were aimed at the recognition of the phenomenon of surface scaling of concrete structures, caused by the action of de-icing salts, were carried out by Arnfelt, Verbeck and Klieger. According to [13], they did not elucidate the mechanisms that cause scaling, but concluded that it is mainly a physical process and that relatively low salt concentrations (2% to 4% by weight) cause the greatest weight loss. The most common de-icing agents are NaCl or CaCl_2_.

A plausible description of the phenomenon was provided by Mather [21], who indicated that the de-icing agent causes snow or ice to melt, with the resulting liquid collecting in “puddles” framed by ice. This liquid is a salt solution and therefore has a lower freezing point. Part of the solution penetrates the concrete and soaks it up at the same time. As the dissolution of the ice proceeds, the solution becomes more and more diluted until its freezing point approaches the freezing point of water. Then the refreezing occurs again. Thus, freezing and thawing may occur as frequently as in the case without the use of de-icing agents.

According to Rösli’s theory [22], high stresses in the surface layer of concrete, consequently leading to peeling of the concrete surface, can be caused by thermal shock when the ice cover is removed by salt action. Calculations presented by him on the basis of laboratory tests indicate that in the case of a 2 mm thick layer of ice and a salt solution (at least 5%), a temperature shock is possible, as a result of which tensile stresses exceeding 4 MPa occur. Rösli’s theory does not explain the case where scaling occurs when the salt solution is already present on the surface and its freezing process occurs.

According to Pühringer [23], who studied the effect of de-icing salts on the frost resistance of cement materials, the detrimental effects of salts are caused by changes in the compressibility of the salt solution compared to water. The primary factor is the greater expansion of an appropriately concentrated salt solution than water at the same temperature, in the case of the thawing process. According to the author of the theory, an increase in salt concentration causes an increase in pressure during the thawing process, while a decrease in salt concentration causes an increase in pressure during the freezing process. When the freezing and thawing processes are cyclic, there is an average value of salt solution concentration that produces the highest stresses. According to calculations made by Pühringer, the highest stresses occur at a concentration of about 2.5% by weight. The calculations involved nine different salts, including those most commonly used as de-icing agents, i.e., CaCl_2_ and NaCl. This is also confirmed by the results of Fagerlund’s research [6], according to whom the destruction of the concrete surface results from the combined action of hydraulic and osmotic pressures. According to Fagerlund’s theory, during the freezing process, ice is first formed in large pores, which leads to hydraulic pressure and is the beginning of the process of osmotic pressure formation. The total pressure in the pore system is directly influenced by the amount and concentration of salt dissolved in solution. According to studies, salt solutions with a concentration of about 3% are the most harmful. This is also confirmed by the results of the studies by Valenza and Scherer [24]. They presented the *glue-spall* mechanism and explained why the scaled material appears as thin flakes. The transport and the crystallization of salt in building materials is the subject of many publications, including [25,26,27].

The phenomenon of the scaling of concrete surfaces in the presence of de-icing agents is different from the volumetric destruction of the concrete structure resulting from freeze and thaw cycles. According to the authors’ experience, and also according to [8,13], the most important factors differentiating the two phenomena can include the following:

- Thermal shock, caused by the temperature difference between concrete and ice treated with de-icing salts;
- Salt concentration and its influence on the freezing process of water;
- Differences in the freezing point of water in the pores of concrete, with and without a salt solution;
- The different characteristics, both physical and chemical, of the surface layer of concrete and inside of it;
- Much stronger effects of external conditions and stresses on the surface of the concrete than on the inside of it;
- Osmotic pressure due to differences between the salt concentration of the solution in the surface layer and in the deeper layers of concrete;
- Effect of de-icing media on the solubility of calcium hydroxide, which has greater solubility in a chloride solution than in water.

**Figure 1:**
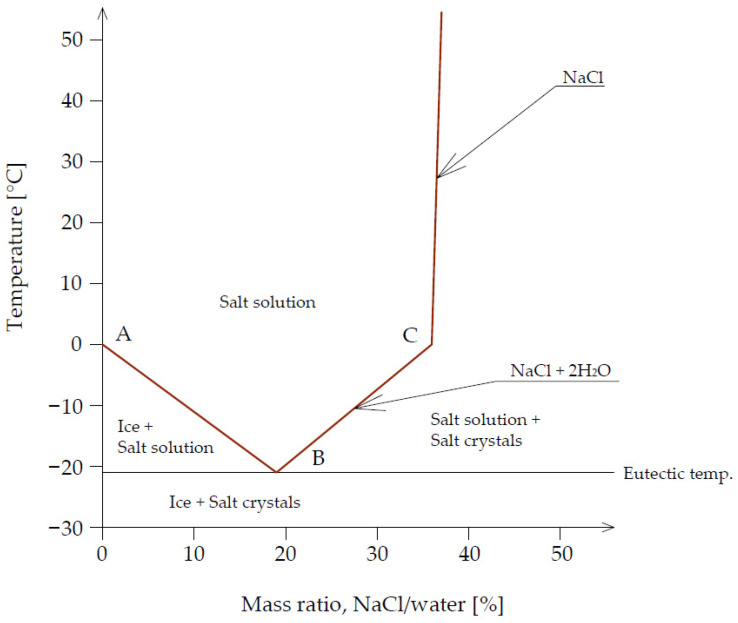
Phase diagram of NaCl solution according to Werse.

The most common de-icing agent is sodium chloride—NaCl. This is due to its widespread availability and lower price, compared to other de-icing agents, such as calcium chloride or magnesium chloride. Based on NaCl and water, the solution is resistant to freezing to a certain temperature, depending on its concentration. Figure 1 shows the phase diagram of the NaCl solution as a function of the solution concentration and its temperature according to Werse, which is described in [28]. The ABC line illustrates the decrease in freezing point of a solution as a function of the solution concentration. The lowest freezing point of a NaCl solution occurs at −21 °C when the concentration of the solution is 28.9%. It should be noted that if sodium chloride is mixed with ice and snow at temperatures above −21 °C, then a salt solution begins to form, and the heat generated is “consumed” in the ice melting process.

To sum up, the phenomenon of surface scaling is a surface erosion phenomenon and usually occurs only when concrete freezes in the presence of de-icing salts. It is a process that is progressive. According to the conducted tests and observations, the phenomenon of the concrete surface scaling depends directly on the concentration of the solution and the number of freeze and thaw cycles. The results of the research by Sellevold and Fastad [29] confirmed that the area of concrete surface damage increases with the extension of the freezing period. The greatest damage occurs when the concentration of the solution is between 2% and 4% and the freezing temperature is no higher than −20 °C.

This is evidenced by the following test methods relating to the frost resistance of concrete with de-icing salts, applied to assess the durability of concrete structures or elements:

- ASTM C 672 standard test method for scaling resistance of concrete surfaces exposed to de-icing chemicals [30], where the top surface of concrete samples, exposed to 3% NaCl solution after 50 cycles of freezing and thawing in the temperature range from −17.8 °C to +23.0 °C, is evaluated;
- SLAB TEST method according to standard PKN-CEN/TS testing—part 9: Freeze–thaw resistance-scaling. The standard refers to three testing methods, among which the SLAB TEST method [31] is a reference method (the upper surface of the sample exposed to 3% NaCl solution is evaluated; the weight of the scaled material after 56 freeze and thaw cycles in the temperature range from −20.0 °C to +20.0 °C is given as the result);
- The method according to PN-EN 1338:2005 [32], where the surface of the sample exposed to 3% NaCl solution is evaluated (loss of weight after 28 freeze and thaw cycles in the temperature range from −20.0 °C to +24.0 °C is given as the result);
- The recommendations of RILEM TC 176-IDC [33].

It should be noted that the phenomenon of surface scaling is much less recognized than the phenomenon of the internal destruction of concrete occurring as a result of cyclic freezing and thawing processes. This is due to the fact that the mechanisms of destruction of the internal structure of concrete are already sufficiently recognized, and the mechanisms of surface scaling are not yet fully understood; in particular, this concerns the mechanism of formation of internal forces at the passage of water into ice and the induction of tensile stresses that destroy the structure of the concrete.

The mechanisms of concrete and mortar damage, caused by cyclic freezing and thawing, are different in the case of internal and external destruction. However, often, only one test method is used to evaluate the material: a test method that simulates the occurrence of internal or external damage due to cyclic freezing and thawing with or without de-icing salts. Therefore, it is reasonable to conduct comparisons of methods simulating the occurrence of both types of damage in order to assess changes in the mechanical properties of mortars.

### 2.2. Test Methods for Repair Mortars with Regard to Frost Resistance According to the PN-EN 1504 Standard

The introduction of the PN-EN 1504 series of standards, including the PN-EN 1504-3 [3] sheet, was aimed, among others, at sorting out and adjusting the scope of tests and requirements for repair materials to the current state of knowledge. According to PN-EN 1504-3 [3], in the testing and evaluation of concrete repair materials, the permanent adhesion to the substrate of the layers made can be evaluated after performing heat compatibility tests. Among the three test methods referred to (see Section 3.2), only the method concerning freeze and thaw cycles with the de-icing salt solution immersion according to PN EN 13687-1 [4] concerns the evaluation of the suitability for use of repair mortars for concrete repair, subjected to frost resistance testing. In the standard [4], the following conditions for performing the test were adopted:

- Temperature changes from −15 ± 2 °C to +21 ± 2 °C;
- Freezing of samples in a saturated NaCl solution at −15 ± 2 °C;
- Thawing of samples in water at +21 ± 2 °C;
- One cycle lasts 4 h and consists of a freezing phase in a saturated NaCl solution for 2 h and the phase of thawing in water for 2 h;
- Tested mortar samples are subjected to shock temperature changes;
- 50 cycles are performed.

On the basis of freeze–thaw cycles carried out with immersion in a de-icing salt solution, only one mechanical property of mortar is determined—mortar adhesion to concrete substrate according to PN-EN 1542 [34], which should be at least equal to the following:

- 1.5 MPa for mortars with compressive strength exceeding 25 MPa (R3 class mortars according to [3]);
- 2.0 MPa for mortars with temporary compressive strength exceeding 45 MPa (R4 class mortars according to [3]).

**Figure 2:**
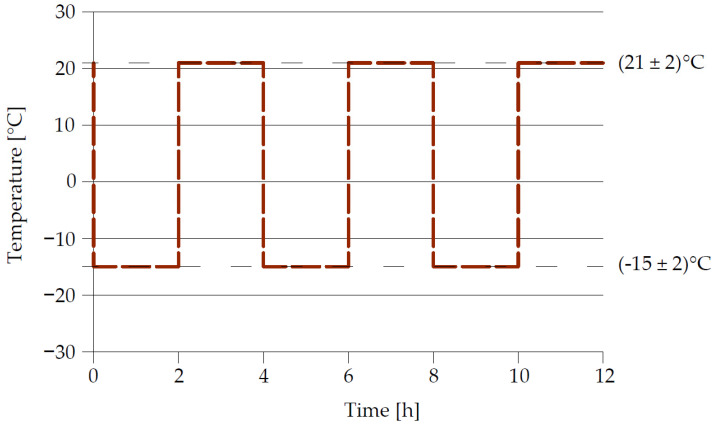
Cycles of freezing in saturated de-icing salt solution and thawing in water, according to [4].

In the case of the determination of resistance to cyclic freezing and thawing by immersion in a de-icing salt solution according to [4], the repair mortar is applied to 3 concrete slabs. Two slabs with applied mortar (test samples) are subjected to the freeze and thaw cycle process of which one is the reference sample. The repair mortar should be applied in accordance with the manufacturer’s instructions, while curing should be carried out in accordance with the conditions given in Annex A of [4]. Before the test, all surfaces of both test samples, except the test surface measuring 300 mm × 300 mm, are sealed with synthetic resin to prevent the penetration of water or saturated NaCl solution from the sides and bottom of the test sample. Test samples prepared in this way, after the seasoning period, are immersed in water for 24 h. The following test cycle is shown in Figure 2.

**Figure 3:**
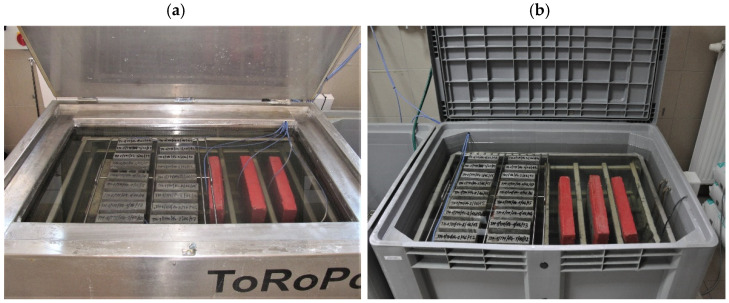
Set of chambers according to [4] (**a**) chamber for testing the freezing of samples in saturated salt solution; (**b**) chamber for testing the thawing of samples in water.

During the test, two specimens are subjected to rapid temperature changes between +21 ± 2 °C and 15 ± 2 °C, including immersion in a saturated, de-icing salt solution, in a special set of test chambers (Figure 3).

After completion of the freezing phase in a chamber filled with a saturated NaCl solution, the samples are washed of salt and placed in a chamber filled with water, where the thawing phase takes place. After the completion of the thawing phase, the samples are removed from the chamber filled with water and placed in a chamber filled with a saturated NaCl solution. Throughout the test cycles, the samples are subjected to shock temperature changes when moving from one test phase to another. After cycles of temperature changes, visible damage is recorded, and adhesion is determined by peeling of the mortar or repair system from the concrete substrate.

The test method for cyclic freezing and thawing by immersion in a saturated de-icing salt solution according to [4] is designed to simulate the process of frost destruction taking place at the external level, with de-icing agents and with a shock temperature change, which manifests itself by surface peeling. It is worth noting that in the PN-EN 1504-3 [3] standard, there are no established test methods for the determination of changes in the strength properties (compressive strength and bending strength) of repair mortars after any frost resistance testing, whether simulating the frost destruction process taking place at the external level or at the internal level. It is also important to note the concentration of the NaCl solution in the study [4], which is almost 30% and many times higher than the concentrations of the NaCl solutions used in other test methods [30,31,32].

### 2.3. Test Methods for Repair Mortars with Regard to Frost Resistance according to National Guidelines

In Poland, a frost resistance test in a climate box has been commonly used for several decades to evaluate the permanent adhesion of concrete repair materials to the substrate and the change of their strength properties. The current test is a modified concrete frost resistance test according to [2] in which the number of cycles of freezing in air and thawing in water is 200. In the standard [2], the following testing conditions for mortars in a climate chamber were adopted:

- Temperature changes from −18 ± 2 °C to +18 ± 2 °C;
- Freezing of water-soaked samples in air but after draining water from the chamber at −18 ± 2 °C;
- Thawing of samples in water at +18 ± 2 °C;
- The freezing cycle in air takes 4 h and the thawing cycle in water from 2 to 4 h.

**Figure 4:**
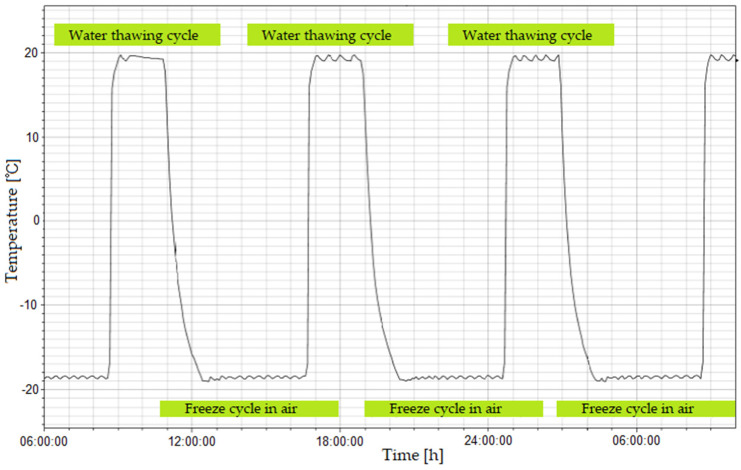
Cycles of freezing in air and thawing in water, according to [18,19].

The course of cycles of freezing in air and thawing in water according to [2] is shown in Figure 4.

Based on the freezing and thawing cycles carried out in the climate box, the following mortar properties are determined:

- Adhesion of the mortar to the concrete substrate by pull-off method, after frost resistance test according to [18], after 200 cycles of freezing in air and thawing in water from −18 ± 2 °C to +18 ± 2 °C;
- Frost resistance (determination of weight loss, decrease in bending strength and decrease in compressive strength) according to [19] after 200 cycles of freezing in air and thawing in water, at temperatures from −18 ± 2 °C to +18 ± 2 °C.

When determining the adhesion of repair mortars to concrete substrate, i.e., their pull-off strength, including after frost resistance testing, mortars are placed on concrete slabs of at least C25/30 class according to [1], with a frost resistance degree of at least F200 according to [2].

Two samples with the repair mortar or the entire repair system applied are prepared for testing. After seasoning, one is subjected to freeze–thaw cycles in a climate box, and the other is stored in a laboratory. The guidelines for the process of the slab freezing and thawing are described in the IBDiM Test Procedure PB/TM-1/13 [18]. After completing the required number of freeze–thaw cycles, usually 200 cycles, the adhesion of the laid layer to the substrate is tested, using the “pull-off” method. The measurement of adhesion to the substrate is performed in accordance with IBDiM Test Procedure PB/TM-1/6 [35] or in accordance with the PN-EN 1542 [34] standard.

The basic criteria for evaluating the adhesion of a tested repair mortar are as follows:

- The average value of temporary adhesion of the repair mortar to the substrate, i.e., the value of adhesion of the material not subjected to frost resistance testing should not be lower than 2.0 MPa (in the case of skimming mortars—1.5 MPa);
- The average value of adhesion to the substrate of the repair mortar subjected to frost resistance testing should not be lower than 1.5 MPa (in the case of skimming mortars—1.2 MPa).

Determination of frost resistance (weight loss, decrease in bending and compressive strength), which allows for evaluating the change in time of the strength properties of the material intended for concrete repair and reprofiling, is described in the IBDiM Test Procedure PB/TM-1/12 [19]. This test is a modified concrete frost resistance test according to standard [2]. Samples in the form of mortar bars with dimensions of 40 mm × 40 mm × 160 mm in the number of 12 pieces are formed from the tested repair materials. After seasoning, all the mortar bars are soaked to a constant weight and then six mortar bars are subjected to a frost resistance test in a climate box, and 6 mortar bars are stored in a bathtub vessel in water. After completing the required number of freeze–thaw cycles, typically after 200 cycles, all mortar bars are weighed to determine weight changes and tested for bending and compressive strength. The criteria for evaluating the frost resistance of repair mortars are as follows:

- The decrease in compressive strength should not exceed 20%;
- The decrease in bending strength should not exceed 20%;
- The loss of weight should not exceed 5%.

Both of the ageing tests outlined above are currently described in National Guidelines WR-M 32 [36] and are referenced in technical specifications for repair mortars. Satisfying the requirements of these tests by repair mortars was the main criterion for granting IBDiM Technical Approvals according to IBDiM Recommendations [37], and now it is the main criterion for granting National Technical Assessments.

## 3. Results

### 3.1. Scope of Comparative Tests

The aim of the project was to compare and analyse the ranges, methodology and results obtained when performing frost resistance tests on repair mortars according to [37], which are currently presented in [36], and frost resistance tests according to [3]. The data obtained as a result of the aforementioned activities carried out within the framework of the project were also to be used for the evaluation of the effectiveness (authoritativeness) of the compared test methods used for performing frost resistance tests. Comparison of the described test methodology for repair mortars cited in [3] and in relation to bridge structures is the subject of several publications including [38,39,40]. This article describes results of comparative tests carried out on six types of repair mortars of the R4 class according to [3]. The comparative study included comparison of the following properties of repair mortars, tested according to the test methods referenced in [3,29]:

- Permanent adhesion, by the pull-off method, of the mortars to the concrete substrate, including surface changes, after the frost resistance test according to [18] and according to [4], with respect to temporary adhesion;
- Frost resistance of mortars (loss of weight and decrease in bending and compressive strength) according to [19] and the modified method according to [19]. The modification of the method [19] was due to the fact that in PN-EN 1504-3 [3], there is no test method given for the determination of frost resistance in the scope of mortar weight loss and decrease in its compressive and bending strength after freeze–thaw cycles. The modification of the method [19] concerned only the course and conditions of the sample freezing and thawing process. Instead of performing 200 cycles of freezing in air and thawing in water at temperatures from −18 ± 2 °C to +18 ± 2 °C, according to [19], 50 cycles of freezing in saturated salt solution at −15 ± 2 °C and thawing in water at +21 ± 2 °C, according to [4], were performed. Due to the fact that in the case of the modified method, according to [19], the main stage of the frost resistance test (freezing and thawing cycles) was performed, according to [4], in the further part of this paper, this test will be referred to as the frost resistance test, according to [4].

### 3.2. Materials and Samples

All repair mortars were of the PCC (Polymer Cement Concrete) type, and their maximum grain size was in the range of 2 to 4 mm. A summary of the repair mortars tested is shown in Table 1.

For all mortars, the mixing ratios were similar. The mixing ratio of the mortar with water was 1.0:0.12–0.15 parts by weight. The method of mixing was in accordance with the manufacturer’s guidelines. Usually, the mortar was stirred with water for 2–4 min, allowed to stand for 3 min and then stirred for about 1 min.

The following samples (objects) were made from each mortar listed in Table 1 for comparative testing:

- Objects of mortar laid on a concrete substrate covered with a bonding layer dedicated by the mortar manufacturer (in the case of all mortars, the bonding layer was cement-based), in the number of four pcs in relation to each mortar, intended to perform an adhesion determination by the pull-off method of the mortar to the concrete substrate, according to the test procedure in [35], including the following:
  – Testing of the object not subjected to the frost resistance test (reference object, so-called witness)—1 pc;
  – Object examination after frost resistance test according to [18] after 200 freeze-in-air and thaw-in-water cycles at temperatures from −18 ± 2 °C to +18 ± 2 °C—1 pc;
  – Testing of objects after the frost resistance test, according to [4], after 50 cycles of freezing in a saturated salt solution at a temperature of −15 ± 2 °C and thawing in water at +21 ± 2 °C—2 pcs.

The number of objects was in accordance with the requirements given in [4,18,35]. The substrate for adhesion testing consisted of concrete slabs made of bridge concrete class C 30/37 of frost resistance grade F200, according to [2]. Considering the influence of the substrate preparation on the results obtained in [41,42,43,44], all substrates were prepared in the same way by mechanical treatment, removing cement laitance and other impurities.

- Mortar objects with dimensions of 40 mm × 40 mm × 160 mm in the quantity of 18 pieces for each mortars, for frost resistance testing:
- According to [19], after 200 cycles of freezing in air and thawing in water at temperatures from −18 ± 2 °C to +18 ± 2 °C;
- According to [4], after 50 cycles of freezing in a saturated salt solution at the temperature of −15 ± 2 °C and thawing in water at +21 ± 2 °C.

As far as the number of objects made was concerned, i.e., 18 objects, 12 were test objects (6 objects for each method) and 6 were reference objects (so called witnesses), common for both of the aforementioned methods.

Curing of the completed test objects was carried out according to the guidelines of the mortar manufacturers. After the curing period, the samples were stored under controlled laboratory conditions at a temperature from 19 to 23 °C and relative humidity of 50 to 70%.

### 3.3. Requirements

To evaluate the obtained results of individual tests of repair mortars carried out as part of comparative tests, the requirements referred to in [37] or [3] were adopted, depending on the test method. Additionally, in the case of the frost resistance test of mortars according to [4], the requirement according to [37] was adopted—the same as in the case of the frost resistance test of mortars, according to [19]. With respect to the determination of adhesion to the concrete substrate by the pull-off method, in the case of permanent adhesion, both in the case of the frost resistance test, according to [4,18], the surface changes that occurred during freezing and thawing cycles were evaluated. A list of the tested mortar properties, including requirements, are shown in Table 2.

### 3.4. Results of Comparative Tests

**Figure 5:**
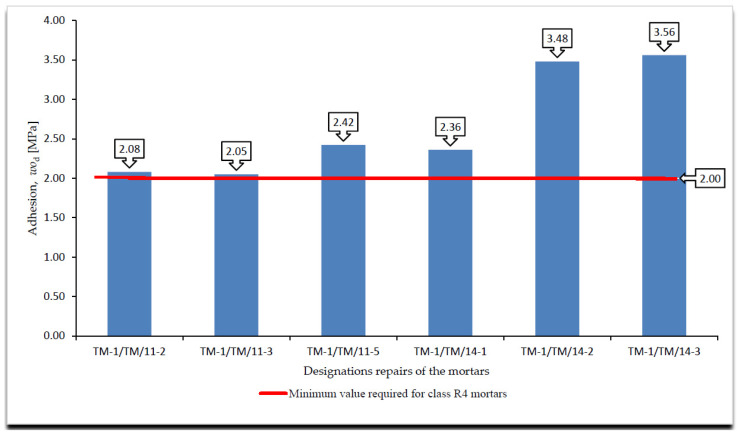
Summary of the average values of temporary adhesion by pull-off method of R4 class mortars to concrete substrate, *wo_d_* [MPa].

**Figure 6:**
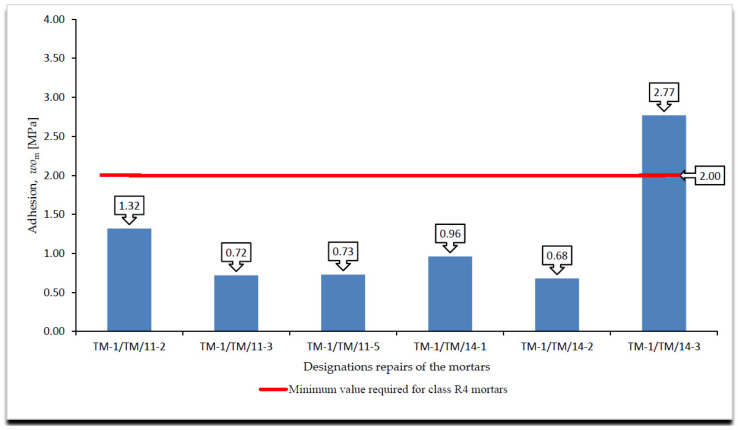
Summary of average values of adhesion by pull-off method for R4 class mortars to concrete substrate after frost resistance test, according to [18], *wo*_m_ [MPa].

**Figure 7:**
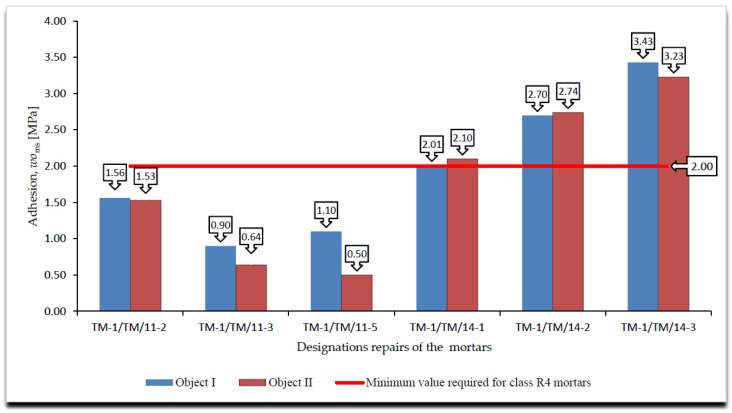
Summary of average values of adhesion by pull-off method for R4 class mortars to concrete substrate after frost resistance test, according to [4], *wo*_ms_ [MPa].

A cumulative summary of the adhesion test results, immediate and permanent after the frost resistance tests, according to [4,18], are presented in Figure 5, Figure 6 and Figure 7, whereas the surface changes that occurred during the freeze and thaw cycles are shown in Table 3.

On the basis of the results of the immediate and permanent adhesion tests after the frost resistance tests, according to [4,18], in the case of the R4 class mortars, the following can be stated in relation to the requirements listed in Table 2:

- All R4 class mortars met the requirements for temporary adhesion to the concrete substrate, i.e., after 28 days of maturing;
- Only one mortar of class R4, designated TM-1/TM/14-3, met the requirements for adhesion to the concrete substrate after the frost resistance test according to [18];
- Three of the six R4 class mortars with designations TM-1/TM/14-1, TM-1/TM/14-2 and TM-1/TM/14-3 met the requirements for adhesion to the concrete substrate after frost resistance test, according to [4];
- In the case of R4 class mortars subjected to the frost resistance test, according to [16], in the case of three mortars with designations TM-1/TM/11-1, TM-1/TM/11-4 and TM-1/TM/11-5, damage on the mortar surface in the form of a grid of cracks was observed. Damage was observed on the surface of mortars that failed to meet the requirement for adhesion to the concrete substrate after the frost resistance test, according to [18];
- In the case of R4 class mortars subjected to the frost resistance test, according to [3], damage was observed on the surface of all the mortars, including mortars with designations TM-1/TM/11-1, TM-1/TM/11-4 and TM-1/TM/11-5. Damage was observed on the surface of the mortars in the form of a grid of cracks, while in the case of the remaining mortars, the near-surface layer was scaled off. Damage in the form of a grid of cracks was observed on the surface of mortars that failed to meet the requirement for adhesion to the concrete substrate after the frost resistance test, according to [4];
- In the case of the occurrence of damage on the mortar surface in the form of a grid of cracks, the beginning of the damage occurred at comparable times of the frost resistance test, e.g., for the mortar designated TM-1/TM/11-2, the crack grid appeared after 100 cycles in the frost resistance test, according to [18], and after 30 cycles in the case of the frost resistance test according to [4], i.e., in approximately half of the duration of both tests;
- Comparing the decreases in permanent versus temporary adhesion after the frost resistance tests, according to [4,18], the greatest difference was observed for the mortar designated TM-1/TM/11-3 for which the decreases in adhesion were the following, respectively:
  – After the frost resistance test, according to [18]—80.45%;
  – After the frost resistance test, according to [4]—21.26% in the case of object I and 22.94% in the case of object II;
- Analysing the types of ruptures during the adhesion tests, using the pull-off method, the following was observed:
  – For mortars not subjected to frost resistance testing for which temporary adhesion was determined, no predominant type of rupture was found but three main types of ruptures were observed: in the concrete substrate, in the bond layer or at the concrete substrate/mortar interface when the mortar was placed directly on the concrete substrate, and partly in the bond layer and partly in the concrete substrate;
  – In the case of mortars subjected to the frost resistance test, according to [18], which failed to meet the requirements concerning adhesion to the concrete substrate, the dominant type of rupture was the rupture between the substrate and the mortar occurring in the bond layer or at the concrete substrate/mortar interface when the mortar was placed directly on the concrete substrate;
  – In the case of mortar TM-1/TM/14-3 tested for frost resistance, according to [18], which met the requirements concerning adhesion to concrete substrate, 60% of ruptures occurred in the concrete substrate and 40% in the bonding layer;
  – For all mortars tested for frost resistance, according to [4], the dominant type of rupture was between the substrate and the mortar in the bond layer or at the concrete substrate/mortar interface when the mortar was placed directly on the concrete substrate;
  – Ruptures in the mortar itself in the determination of permanent adhesion occurred only in the case of mortars subjected to the frost resistance test, according to [4], and they accounted for only 10% of all types of ruptures, with only 20% of these ruptures being 100% in the mortar.
- The joining of the mortar with the concrete substrate was the weakest element in the samples tested for frost resistance, according to [4,18], although in all cases, the bonding layer was present to increase the adhesion of the mortar to the concrete substrate;
- Comparing the obtained results of the permanent adhesion of R4 class mortars subjected to frost resistance tests, according to [4,18], a greater decrease in adhesion was observed in the case of mortars subjected to the frost resistance test according to [18], although in the case of the frost resistance test according to [4], damage was recorded on all surfaces of the tested mortars;
- In the case of mortars subjected to the frost resistance test, according to [18], it was observed that not in all cases the decrease in permanent adhesion is connected with the occurrence of damage on the mortar surface. This was due to the fact that the weakest element in the tested samples was the bond between the mortar and the concrete substrate;
- According to the author, one of the decisive factors affecting the greater decrease in adhesion occurring in the mortars subjected to the frost resistance test is how the sample is secured. In the case of performing the frost resistance test, according to [4], the test sample is covered from the bottom and on the sides with synthetic resin, which forms a tight coating, preventing the penetration of the saturated NaCl solution between the mortar and the concrete substrate.

**Figure 8:**
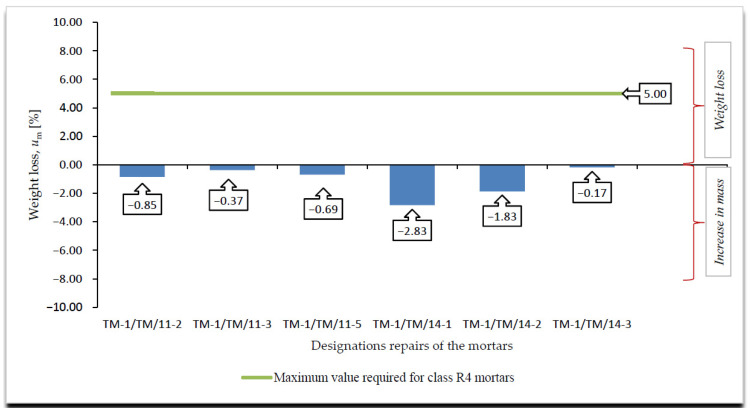
Summary of averages values of weight loss of R4 class mortars after frost resistance testing, according to [19], *u*_m_ [%].

**Figure 9:**
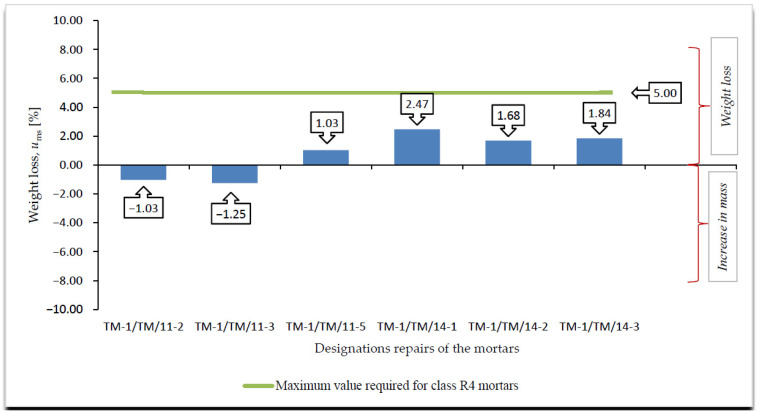
Summary of averages values of weight loss of R4 class mortars after frost resistance testing according to [4], *u*_ms_ [%].

**Figure 10:**
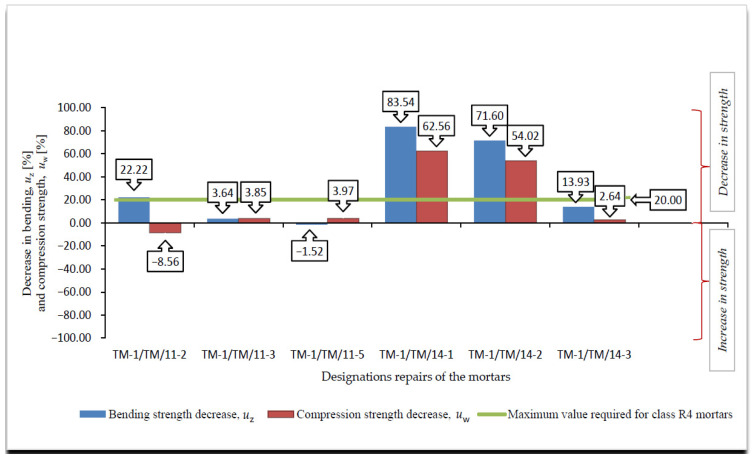
Summary of average values of decrease in bending *u*_z_ [%] and compression strength *u*_w_ [%] of R4 class mortar after frost resistance testing, according to [19].

**Figure 11:**
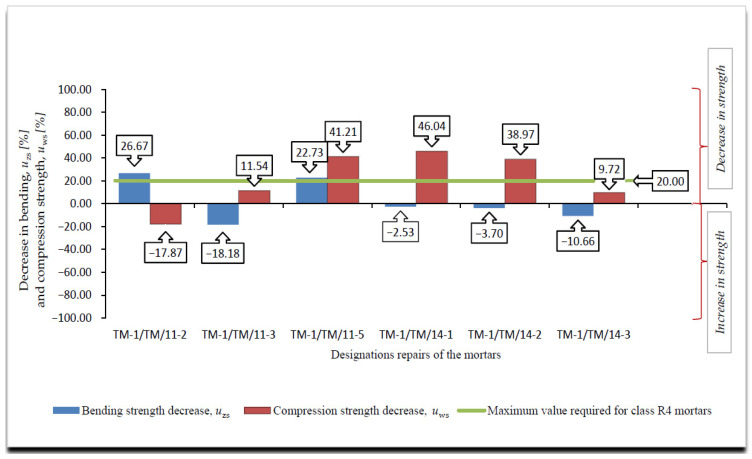
Summary of average values of decrease in bending *u*_zs_ [%] and compression strength *u*_ws_ [%] of R4 class mortars after frost resistance testing, according to [4].

A summary of the results of the frost resistance tests (weight loss, decrease in bending and compressive strength), according to [4,19], for R4 class mortars are presented in Figure 8, Figure 9, Figure 10 and Figure 11.

**Figure 12:**
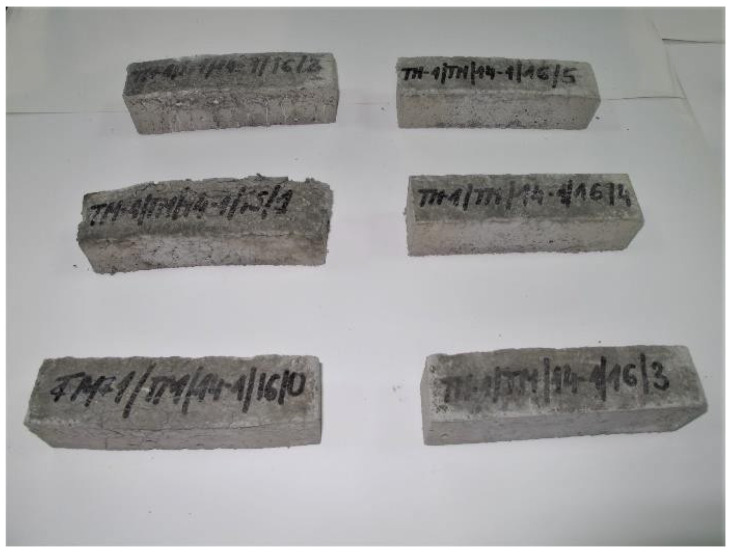
View of TM-1/TM/14-1 repair mortar samples after freeze-thaw cycles according to [19]. Visible deformation, cracks and weight loss of the samples, especially with the following designations: TM-1/TM/14-1/16/0, TM-1/TM/14-1/16/1 and TM-1/TM/14-1/16/2.

On the basis of the results of frost resistance tests carried out in accordance with [4,19], in the case of R4 class mortars, it can be stated that in relation to the requirements listed in Table 2:

- Three R4 class mortars with designations TM-1/TM/11-3, TM-1/TM/11-5 and TM-1/TM/14-3 met the requirements of the frost resistance test, according to [19];
- Two R4 class mortars with designations TM-1/TM/11-3 and TM-1/TM/14-3 met the requirements of the frost resistance test, according to [4];
- Mortars that met the frost resistance test requirements according to [4], also met the frost resistance test requirements according to [19];
- In the case of the frost resistance test according to [19], an increase in the weight was observed for all mortars, which is typical of the frost resistance test carried out according to [17], and indicates the occurrence of micro scratches into which water penetrates and causes an increase in the weight of samples, which, in consequence, often leads to frost damage in the form of deformations, cracks, and weight loss (Figure 12);
- R4 class mortars that fulfilled the requirements of the frost resistance test according to [19] were characterised by the lowest increase in weight after cycles of freezing in air and thawing in water, i.e., TM-1/TM/11-3—0.37%, TM-1/TM/11-5—0.69% and TM-1/TM/14-3—0.17%;
- In the case of most mortars (four out of six mortars) after frost resistance testing, the decrease in bending strength was also accompanied by a decrease in compressive strength. In the case of R4 class mortar designated TM-1/TM/11-2, a significant increase in compressive strength was observed at 8.56%, despite a high value of decrease in bending strength at 22.22% (above the required level);
- In the case of the frost resistance test according to [4], an increase in the mass was observed in relation to the two mortars designated TM-1/TM/11-2 and TM-1/TM/11-3, whereas in relation to the other mortars, a decrease in mass was observed, the highest in relation to the mortar designated TM-1/TM/14-1, at 2.47%;
- For the mortar designated TM-1/TM/14-1, after the frost resistance testing according to [4], the highest decrease in compressive strength was also observed at 46.04%;
- Only in the case of one mortar, designated TM-1/TM/14-5, after the frost resistance testing, a simultaneous decrease in compressive and bending strengths was observed;
- In the case of as many as four mortars after frost resistance testing, according to [4], despite a decrease in compressive strength—including in the two mortars designated TM-1/TM/14-1 and TM-1/TM/14-2 for which there was a decrease of 46.04% and 38.97%, respectively—an increase in bending strength was found;
- In the case of PCC mortars, the mortar’s bending strength properties are mainly shaped by adding appropriate modifiers, whereas in the case of compressive strength, they are often shaped only by using an appropriate grade of cement and selecting a suitable aggregate composition. The results obtained confirm the positive influence of the modifiers on the mortar properties with respect to bending strength.

To summarize, the results obtained for the repair mortars were in terms of the following:

- Permanent adhesion, by the pull-off method, of the mortars to the concrete substrate, including surface changes, after the frost resistance test, according to [4,18], with respect to temporary adhesion;
- Frost resistance of mortars (loss of weight and decrease in bending and compressive strength), according to [4,19].

**Figure 13:**
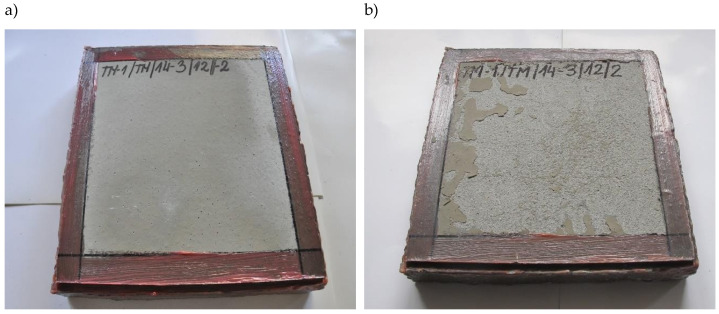
View of mortar sample TM-1/TM/14-3 (object II); (**a**) before the frost resistance test according to [4], and (**b**) after the frost resistance test according to [4].

Therefore, the following can be concluded:

- All R4 class mortars met the requirements regarding of temporary adhesion to the concrete substrate;
- Only one mortar, designated TM-1/TM/14-3, met the requirements of temporary and permanent adhesion, according to [4,18], and the frost resistance tests, according to [4,19], whereby the frost resistance test according to [4] was followed by complete scaling of the surface layer (Figure 13);
- In the case of the test of the permanent adhesion of mortars after the frost resistance test according to [18], greater decreases in the average value of adhesion were recorded than in the case of the test of the permanent adhesion of mortars after the frost resistance test according to [4]. The largest average decrease in adhesion values was observed in case of the R4 class mortar with designation TM-1/TM/14-2 at 2.8 MPa;
- In the case of the designation of permanent adhesion, according to [4,18], the dominant type of rupture in the pull-off test was the rupture between the substrate and the mortar in the bond layer. For this type of rupture, it could be concluded that the tensile strength of the mortar was higher than the measured values;
- The frost resistance testing method according to [19] gives results that are easier to interpret than the frost resistance testing method according to [4]. In the case of the frost resistance testing method according to [19], a decrease in compressive strength was usually accompanied by a decrease in bending strength, whereas if there was an increase in the bending strength of the mortar with a simultaneous decrease in the compressive strength of the same mortar, or vice versa, it was less than 10%;
- In the frost resistance testing method according to [4], significant differences were found in the changes of the tested properties (weight loss, decrease in bending strength and decrease in compressive strength). Significant increases in the bending strength of the mortar were observed with a decrease in the compressive strength of the same mortar or vice versa;
- Maximum differences in the case of the frost resistance test according to [4] were observed in relation to mortars designated TM-1/TM/14-2 and TM-1/TM/14-3, where, respectively, in the case of the mortar designated TM-1/TM/14-2, the decrease in bending strength by 26.67% was accompanied by an increase in compressive strength by 17.87%. In the case of the mortar designated TM-1/TM/14-3, the increase in bending strength by 18.18% was accompanied by a decrease in compressive strength by 11.54%.

## 4. Discussion and Conclusions

Based on the analysis of the comparative results obtained, the following can be concluded:

- No mortar met the requirements, according to [37] (currently presented in [36]) and [3] listed in Table 2;
- Only two mortars, marked TM-1/TM/11-1 and TM-1/TM/14-3, met the requirements according to [28];
- No mortar met the requirements according to [3];
- In the case of temporary or permanent adhesion of the mortar to the concrete substrate, the weakest element of the mortar–concrete substrate system is the connection between the mortar and the concrete substrate;
- Comparing the results of the permanent adhesion of R4 class mortars subjected to the frost resistance tests according to [4,18], it was observed that a greater decrease in adhesion occurred in the case of samples subjected to the frost resistance test according to [18];
- In the case of all tested mortars subjected to the frost resistance test according to [4], damage was found on their surface;
- In the case of half of the tested R4 class mortars, the occurrence of damage on the surface after the frost resistance test according to [4] did not cause the permanent adhesion to decrease below the required value;
- In the case of frost resistance tests according to [4,18], the conformity of changes was found only in the case of compressive strength changes; in the case of weight and bending strength changes, opposite directions of changes were found;
- The frost resistance testing method according to [18] gives results that are easier to interpret than the frost resistance testing method according to [4]. In the case of the frost resistance testing method according to [18], a decrease in compressive strength was usually accompanied by a decrease in bending strength, while if there was an increase in the bending strength of the mortar with a corresponding decrease in the compressive strength of the same mortar, or vice versa, it was usually insignificant;
- In the case of the method according to [4], significant differences were found in the changes of the properties tested (weight loss, decrease in bending strength and decrease in compressive strength). Significant increases in the bending strength of the mortar were observed with a corresponding decrease in the compressive strength of the same mortar or with reverse changes. This phenomenon complicates the interpretation of frost resistance assessment according to [4].

The tests carried out also showed that when evaluating mortars for concrete repair, the determination of permanent adhesion to the substrate of the layers made from them and the change in strength properties can be carried out after frost resistance testing in a climate box. The main problem is the selection of the right procedure for frost resistance testing in the climate box. According to the authors, based on the observations made during the conducted research and the analysis of the obtained results, the methods referred to in [36] are more authoritative, as well as being more comprehensive, for the assessment of the durability and effectiveness of the repair of bridge structures using mortars. The observations made by the authors in relation to repair mortars used on bridges indicate that the main reason for failure to ensure the effectiveness of a repair is not the scaling of the mortar surface layer, but the lack of permanent adhesion or the lack of adequate strength properties. Additionally, in the case of repair mortars, in order to obtain a uniform appearance of the repaired element, protective coatings are applied, or surface protection is carried out by means of water repellence, which, to a large extent, limits the possibility of penetration of de-icing agents, including NaCl, into the mortar structure. According to the authors, mortar ageing tests, in this case, frost resistance, should be consistent with the ageing tests of the concrete substrate. Corrosion processes in concrete structures caused by chlorides are often the main factors that have a negative impact on the durability of the structures [9,43,45,46]; therefore, the method of testing the frost resistance of mortars used to repair concrete according to [3] should be consistent with the applied methods of concrete frost resistance tests according to [30,31,32] because only then will we be able to correctly assess the resistance of the repaired structure to aggression caused by cyclic freezing and thawing with the use of de-icing salts by means of ageing tests.

In the case of concrete, the most frequently performed test on the Polish market is the frost resistance test according to [2], which makes it possible to determine the loss of weight and the decrease in compressive strength. Currently, it is standard practice to test concrete according to [2] to meet the requirements for frost resistance grade F200. Therefore, in our opinion, the frost resistance test according to [3] is not sufficient for proper evaluation of repair mortars in terms of aggression caused by cyclic freezing and thawing.

According to the authors, it is also very important to determine the bending strength of repair mortars. This applies to both the temporary bending strength and the after-frost resistance test. One of the main purposes of modifying mortars with polymers was to improve their properties in this respect. According to the authors, the lack of a testing method in PN-EN 1504-3 [3] that allows to verify this property is, to a large extent, a negation of one of the main purposes of mortar modification.

## Figures and Tables

**Table 1:** Summary of repair mortars subjected to comparative tests.

No.	Designation Repair Mortar ^(1)^	Grain Size, (mm)	Compressive Strength ^(2)^, (MPa)	Class as per PN-EN 1504-3 [3]	Bonding Layer
1	TM-1/TM/11-2	≤4	≥55	R4	yes
2	TM-1/TM/11-3	≤2	≥45	R4	yes
3	TM-1/TM/11-5	≤4	≥45	R4	yes
4	TM-1/TM/14-1	≤2	≥55	R4	yes
5	TM-1/TM/14-2	≤4	≥55	R4	yes
6	TM-1/TM/14-3	≤2.5	≥80	R4	yes

^(1)^ Internal management system designation assigned to samples intended for testing. ^(2)^ Compressive strength as declared by the manufacturer.

**Table 2:** List of tested mortar properties, including requirements.

No.	Property	Requirement According to [37]	Requirement According to [3]
1	Temporary adhesion (average), by pull-off method, of the mortar to the concrete substrate, *wo_d_* [MPa]	≥2.0	≥2.0
2	Adhesion (average), by pull-off method, of mortars to concrete substrates; after frost resistance test according to [18], *wo_m_* [MPa]	≥2.0	-
	Surface changes after frost resistance test according to [18]	No change	-
3	Adhesion (average), by the pull-off method, of the mortar to the concrete substrate; after frost resistance test according to [4], *wo_ms_* [MPa]	-	≥2.0
	Surface changes after the frost resistance test according to [4]	-	No change
4	Frost resistance according to [18]	-	-
	-Loss of weight, *u_m_* [%]	≤5	-
	-Decrease in bending strength, *u_z_* [%]	≤20	-
	-Decrease in compressive strength, *u_w_* [%]	≤20	-
5	Frost resistance according to [4]	-	-
	-Weight loss, *u_ms_* [%]	-	≤5 ^(1)^
	-Decrease in bending strength, *u_zs_* [%]	-	≤20 ^(1)^
	-Decrease in compressive strength, *u_ws_* [%]	-	≤20 ^(1)^

^(^^1)^ No reference to a test method in [3], the requirement according to [37] was adopted.

**Table 3:** Summary of results of surface changes in R4 class mortars after frost resistance testing, according to [4,16].

No.	Property	Test Result	Value Required
R4 class mortar with designation TM-1/TM/11-2			
1	Surface changes after the frost resistance test according to [18]	After 100 cycles, there was a grid of cracks on the mortar surface	no changes
	Surface changes after the frost resistance test according to [4]:	-	-
	-object I	After 30 cycles, there was a grid of cracks on the mortar surface	no changes
	-object II	After 30 cycles, there was a grid of cracks on the mortar surface	no changes
R4 class mortar with designation TM-1/TM/11-4			
2	Surface changes after the frost resistance test according to [18]	After 150 cycles, there was a grid of cracks on the mortar surface	no changes
	Surface changes after the frost resistance test according to [4]:	-	-
	-object I	After 40 cycles, there was a grid of cracks on the mortar surface	no changes
	-object II	After 40 cycles, there was a grid of cracks on the mortar surface	no changes
R4 class mortar with designation TM-1/TM/11-5			
3	Surface changes after the frost resistance test according to [18]	After 175 cycles, there was a grid of cracks on the mortar surface	no changes
	Surface changes after the frost resistance test according to [4]	-	-
	-object I	After 40 cycles, there was a grid of cracks on the mortar surface	no changes
	-object II	After 40 cycles, there was a grid of cracks on the mortar surface	no changes
R4 class mortar with designation TM-1/TM/14-1			
4	Surface changes after the frost resistance test according to [18]	no changes	no changes
	Surface changes after the frost resistance test according to [4]:	-	-
	-object I	After 30 cycles, there was a process of scaling of the mortar surface	no changes
	-object II	After 30 cycles, there was a process of scaling of the mortar surface	no changes
R4 class mortar with designation TM-1/TM/14-2			
5	Surface changes after the frost resistance test according to [18]	no changes	no changes
	Surface changes after the frost resistance test according to [4]:	-	-
	-object I	After 30 cycles, there was a process of scaling of the mortar surface	no changes
	-object II	After 30 cycles, there was a process of scaling of the mortar surface	no changes
R4 class mortar with designation TM-1/TM/14-3			
6	Surface changes after the frost resistance test according to [18]	no changes	no changes
	Surface changes after the frost resistance test according to [4]:	-	-
	-object I	After 20 cycles, there was a process of scaling of the mortar surface	no changes
	-object II	After 20 cycles, there was a process of scaling of the mortar surface	no changes

## Data Availability

Data are contained within the article.
